# Motility Assessment of Ram Spermatozoa

**DOI:** 10.3390/biology11121715

**Published:** 2022-11-26

**Authors:** Madeleine Van de Hoek, Jessica P. Rickard, Simon P. de Graaf

**Affiliations:** Faculty of Science, The University of Sydney, Sydney, NSW 2006, Australia

**Keywords:** flagellar, metabolism, morphology, motion, ovine, quality, sperm

## Abstract

**Simple Summary:**

This review describes the mechanical and energetic processes which underpin mammalian sperm motility as well as the mechanisms by which various agents influence the motility of ram spermatozoa. Furthermore, current methods of motility assessment used, or intended for use, in the sheep livestock production industry are explored in relation to ram semen quality control and prediction of artificial insemination success. Such methods include mass motility assessment, subjective motility evaluation and computer-assisted sperm analysis as well as newly developed methods of motility assessment which enable analysis of a new range of sperm motion parameters, including flagellar tracing, three-dimensional sperm tracing, in vivo motility assessment and molecular assays. Improving the understanding of sperm motility and the methods used in its assessment will facilitate optimisation of ram semen processing and quality assessment for use in ovine artificial breeding programs.

**Abstract:**

For successful fertilisation to occur, spermatozoa need to successfully migrate through the female reproductive tract and penetrate the oocyte. Predictably, poor sperm motility has been associated with low rates of fertilisation in many mammalian species, including the ram. As such, motility is one of the most important parameters used for in vitro evaluation of ram sperm quality and function. This review aims to outline the mechanical and energetic processes which underpin sperm motility, describe changes in motility which occur as a result of differences in sperm structure and the surrounding microenvironment, and assess the effectiveness of the various methods used to assess sperm motility in rams. Methods of subjective motility estimation are convenient, inexpensive methods widely used in the livestock industries, however, the subjective nature of these methods can make them unreliable. Computer-assisted sperm analysis (CASA) technology accurately and objectively measures sperm motility via two-dimensional tracing of sperm head motion, making it a popular method for sperm quality assurance in domesticated animal production laboratories. Newly developed methods of motility assessment including flagellar tracing, three-dimensional sperm tracing, in vivo motility assessment, and molecular assays which quantify motility-associated biomarkers, enable analysis of a new range of sperm motion parameters with the potential to reveal new mechanistic insights and improve ram semen assessment. Experimental application of these technologies is required to fully understand their potential to improve semen quality assessment and prediction of reproductive success in ovine artificial breeding programs.

## 1. Introduction

The ability of spermatozoa to successfully migrate through the female reproductive tract and subsequently, penetrate the oocyte is essential for fertilisation success. Following either artificial insemination or natural mating, ram spermatozoa must navigate through the complex fluidic and biochemical environment of the ewe reproductive tract including the: vagina and cervix (unless bypassed by artificial means), uterus, uterotubal junction and fallopian tubes (oviduct) [1]. As such, sperm motility is one of the most important parameters used for quality evaluation. Sperm motility is defined as a propagation of transverse waves along the flagellum in a proximal-distal direction which produces an impulse that pushes the spermatozoon progressively forward through the female reproductive tract [2]. Predictably, poor sperm motility has been associated with low rates of fertilisation success in many species [3,4,5,6,7,8].

To accurately assess sperm motility in vitro, it is essential to understand factors which alter motility. Differences in the morphological structure of spermatozoa, such as those evident between mammalian species, appear to impose differences in sperm metabolism and mechanics which in turn influence flagellar motion and sperm velocity [9,10]. In addition, processes which inflict morphological and ultrastructural damage onto spermatozoa reduce motility via interference with the cell’s metabolism and energy production pathways [11,12]. Throughout the sperm journey in the female reproductive tract, spermatozoa undergo maturation processes, including capacitation and hyperactivation, which significantly alter flagellar motion [13]. These changes in motility are theorised to improve the effectiveness of their migration through the female reproductive tract and assist in enabling successful fertilisation [1]. Furthermore, the properties of the fluidic and biochemical environment which spermatozoa encounter throughout their journey to fertilisation, including temperature, pH and osmolarity, and medium viscosity, appear to further influence sperm motility [12,14,15].

Mass motility assessment and microscopic motility estimation are convenient, inexpensive methods of motility assessment widely used in industry. Mass motility assessment assesses the three-dimensional collective wave motion of a group of spermatozoa, while microscopic motility evaluation involves making a visual estimation of the percentage of individual progressively motile spermatozoa in an ejaculate. Mass motility, in particular, is useful in ram semen motility assessment due to its correlation to in vivo sperm fertilising capacity [16]. However, the subjective nature of these methods limit precision and repeatability, risking potential unreliability when it comes to male fertility assessment [17,18]. By subjective we mean that the motility score is determined by the technician and thus impacted by individual interpretation. Alternatively, computer-assisted sperm analysis (CASA) technology widely used across industry, provides an objective assessment of sperm motility [19]. By objective we mean that the motility score is determined by technology and thus standardised across individual technicians and laboratories. Multiple CASA motility parameters, including curvilinear velocity, average path velocity and beat cross frequency, were correlated with rams with high in vivo fertility [20,21,22]. As such, CASA motility analysis is considered useful for providing information important for quality assessment of semen, and for studying the effect of changes in the microenvironment on specific sperm motion characteristics in vitro. However, despite the sperm flagellum being the primary driver of sperm motility in a three-dimensional manner, current CASA systems assess motility exclusively via tracing of sperm head motion in two dimensions [23]. It has therefore been argued that CASA motility analysis lacks the mechanistic insight necessary to give a true assessment of sperm motility parameters. Development of new motility assessment technologies, such as flagellar tracing, three-dimensional tracing systems, in vivo motility assessment and molecular assays which quantify motility-associated biomarkers, have been proposed as complimentary assessment methods which enable analysis for a new range of sperm motion parameters beyond those that traditional motility assessment techniques provide. The patterns of spermatozoa kinematics detected and quantified by these methods may provide a more detailed insight into the subtle changes in sperm movement which occur in response to the physical and biochemical environment, which have a significant impact on overall sperm motility.

This review describes the mechanical and energetic processes which occur in mammalian spermatozoa to drive motility. In addition, the mechanisms by which various agents influence the motility of spermatozoa, including morphology, cell damage, maturation and its surrounding environment, will be discussed. Finally, current methods of motility assessment used, or intended for use in the sheep livestock production industry, will be explored in relation to ram semen quality control and prediction of artificial insemination success. Improving the understanding of sperm motility and the methods used in its assessment will facilitate optimisation of semen processing and quality assessment for use in ovine artificial breeding programs.

## 2. Structure of Spermatozoa

**Figure 1:**
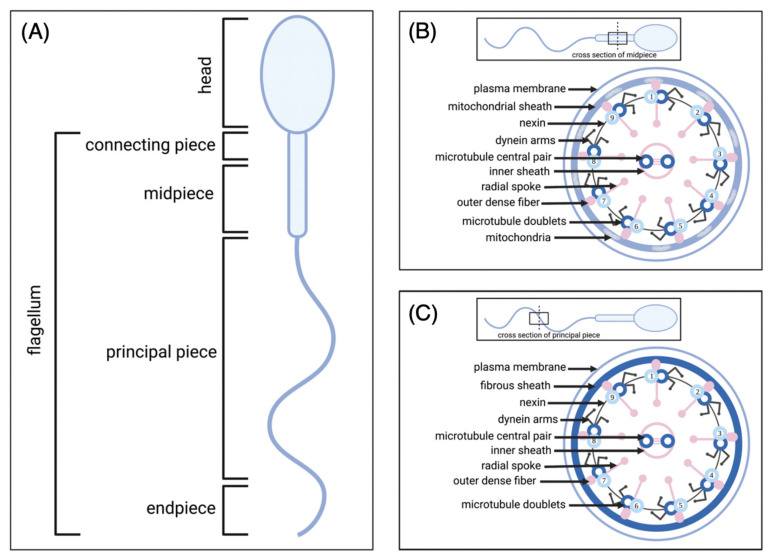
(**A**) mammalian spermatozoon structure. Spermatozoon are divided into two main structures: the head and the flagellum. The flagellum is further divided into four ultrastructures: connecting piece, midpiece, principal piece, and endpiece; (**B**) cross-section of the flagellum structure in the midpiece containing the axoneme surrounded by outer dense fibers, mitochondrial sheath and the plasma membrane; (**C**) cross-section of the flagellum structure in the principal piece containing the axoneme surrounded by outer dense fibers, fibrous sheath and the plasma membrane. Figures created with BioRender.com (accessed on 21 November 2022).

Understanding the structure of a spermatozoon is an essential first step in analysing motility. Mammalian spermatozoa are composed of two main structures: the head and the flagellum (Figure 1A). The sperm head is comprised of the nucleus and the acrosome, containing the cells genetic information (in the form of haploid DNA) to be delivered to the oocyte, and its membrane comprising numerous surface receptors and proteins [24]. The flagellum contains the motile apparatus necessary for inducing and regulating sperm motility. It is responsible for generating the force for flagellar movement [2], responding to changes in viscosity [25], enabling rheotactic behaviour [26], and cell guidance and navigation through boundary sensing [27]. The flagellum can be further divided into four ultrastructures (Figure 1A). The connecting piece attaches the flagellum to the sperm head, the midpiece contains sperm mitochondria, and the principal piece and the end piece together generate the flagellar waveform [28].

**Figure 2:**
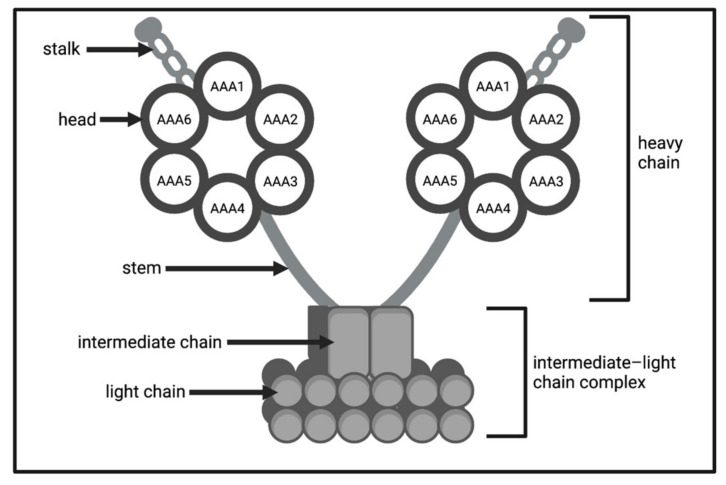
Outer dynein arm structure. Outer dynein arms consist of an an intermediate–light chain complex, along with heavy chain structures each composed of a head, a stalk and a stem [31]. The stem binds the intermediate–light chain complex to the head [38]. The head is an ATPase associated (AAA) ring which consists of six AAA domains (AAA1-6). AAA1 is the primary ATPase site responsible for the hydrolysis of ATP [38]. The function of the remaining AAA domains (AAA2-6) is largely unknown however it is thought that they are important for structure and regulation of dynein movement [39]. The stalk is connected to the head and has a microtubule binding domain at its tip [38].

The primary structure in the flagellum is the axoneme (Figure 1B,C). This structure begins in the connecting piece, terminates in the endpiece, and consists of a central pair of microtubules surrounded by nine pairs of microtubule doublets (arranged concentrically around the central pair in a 9 + 2 structure). Microtubule doublets are connected to adjacent doublets and the central microtubule pair by nexin links and radial spokes, respectively [24]. Attached to microtubule doublets are dynein arms (inner and outer), regulators, and other axonemal complexes which are spatially organized into 96 nm repeats along the axoneme [29,30]. Each axonemal repeat contains 11 dynein complexes that form two distinct rows of outer dynein arms and inner dynein arms, respectively, along the length of each microtubule doublet [30]. Dyneins are multisubunit motor enzymes, made up of several heavy, intermediate and light chains, which together hydrolyse adenosine triphosphate (ATP) and use the energy to cause movement of the microtubules [31]. Outer dynein arms (Figure 2) consist of an an intermediate–light chain complex, along with heavy chain structures each composed of an ATPase associated (AAA) ring (head), a stalk and a stem [31].

Mammalian spermatozoa have accessory structures located between the axoneme and the plasma membrane, which differ slightly between each flagellar ultrastructure. In the midpiece, the axoneme is surrounded by outer dense fibers and the mitochondrial sheath, attached together by a reticulum of filaments (Figure 1B) [32]. The mitochondrial sheath is composed of mitochondria coiled helically around the axoneme, responsible for the generation of ATP for the sperm cell via a process of oxidative phosphorylation [33,34]. Although their main role surrounds energy generation and regulation of metabolism, mitochondria are also involved in the regulation of redox equilibrium, calcium homoeostasis and apoptotic pathways which are necessary for sperm motility and function [35]. In the principal piece the axoneme is surrounded by outer dense fibers and fibrous sheath, enriched with glycolytic enzymes (Figure 1C). In the fibrous sheath, energy is generated via a process of glycolysis.

Along the flagellum of mammalian sperm, there are numerous structural and signalling proteins which are involved in the initiation and regulation of sperm motility. These include guanosine 3′,5′-cyclic monophosphate (cGMP)-dependent potassium channels, cyclic adenosine monophosphate (cAMP)-dependent protein kinase channels, various calcium channels, calcium-ATPase pumps, sodium bicarbonate cotransporters (Na^+^/HCO_3_^−^) and sodium-hydrogen exchangers (Na^+^/H^+^) [29,36]. Additionally, structural proteins in the fibrous sheath including AKAP3 and AKAP4, among others, interact with protein partners in these pathways (kinases, phosphatases, and glycolytic enzymes) to regulate sperm motility [37]. The specific roles and regulatory mechanisms of each of these pathways will be discussed later in the review.

## 3. Movement of Spermatozoa

### 3.1. Energy Requirements

Sperm motility is dependent on energy availability. ATP serves as the primary energy source used by axonemal dynein ATPases within the flagellum to induce a flagellar beat [40]. ATP in spermatozoa is generated using a combination of metabolic pathways, including oxidative phosphorylation and the Krebs cycle in the mitochondria of the midpiece and, glycolysis in the principal piece of the flagellum.

**Figure 3:**
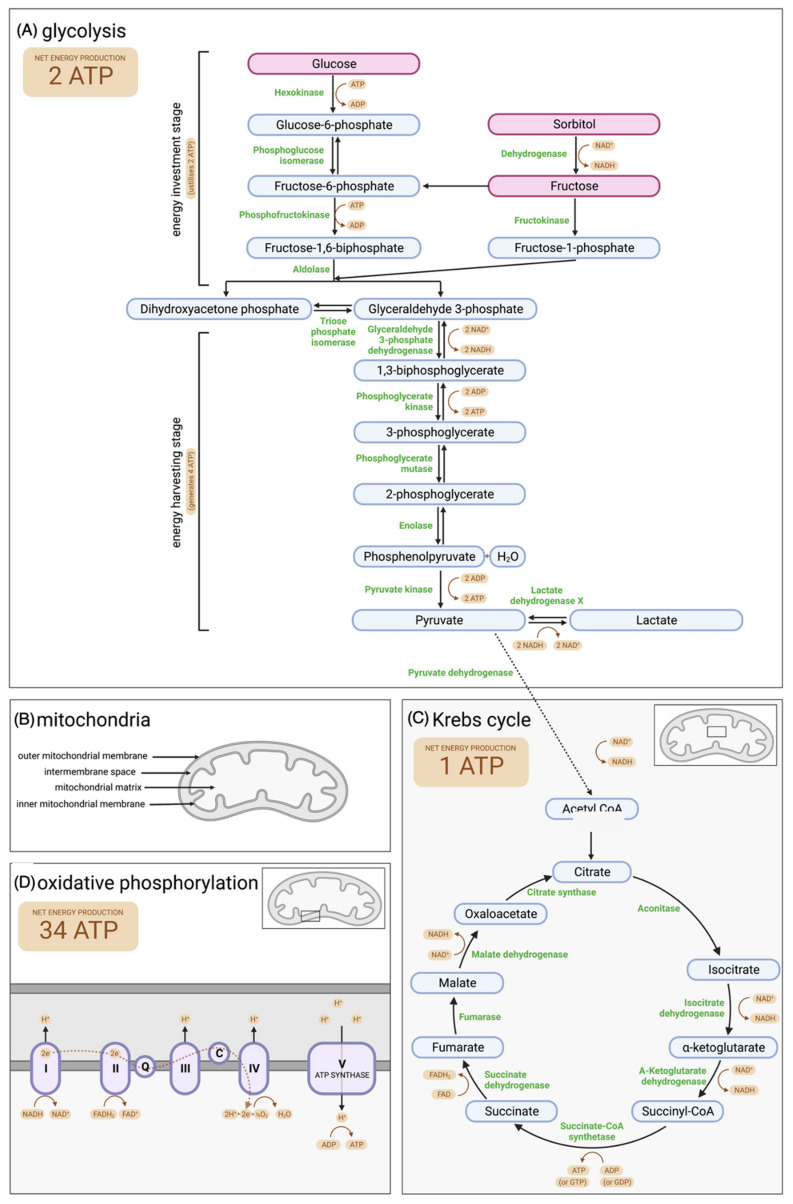
(**A**) Glycolysis. Occurs in the principal piece of spermatozoa. During glycolysis glucose, fructose or sorbitol is converted into pyruvate with a net gain of 2 adenosine triphosphate (ATP) molecules. Each step of glycolysis is catalysed by sperm-specific isoenzymes found in the principal piece, shown in green [42]. Glycolysis is broken into two phases: the energy investment stage and the energy harvesting stage. The energy investment stage utilises 2 molecules of ATP to convert glucose into glyceraldehyde 3-phosphate via glucose-6-phosphate, fructose-6-phosphate and fructose-1,6-biphosphate. Alternatively sorbitol or fructose can enter the glycolytic pathway after being converted into either fructose-6-phosphate or fructose-1-phosphate. The energy harvesting stage involves the conversion of glyceraldehyde 3-phosphate into pyruvate via 1,3-biphosphoglycerate, 3-phosphoglycerate, 2-phosphoglycerate and phosphoenolpyruvate. This stage produces 4 molecules of ATP. (**B**) Mitochondria. Found helically coiled around the midpiece within the mitochondrial sheath [34]. Mitochondria consist of an outer and inner membrane which delimit the intermembrane space and the mitochondrial matrix [35]. Energy generation occurs here in the form of the Krebs cycle and oxidative phosphorylation. (**C**) The Krebs cycle (citric acid cycle or tricarboxylic acid cycle). Occurs in the mitochondrial matrix catalysed by sperm-specific isoenzymes, shown in green [42]. Pyruvate generated via glycolysis can be imported through specific transporters into the mitochondria and oxidised into acetyl coenzyme A (acetyl CoA). Acetyl CoA then enters the Krebs cycle which generates nicotinamide adenine dinucleotide (NADH) from NAD+, flavin adenine dinucleotide (FADH_2_) from FAD+ and 1 molecule of either guanosine triphosphate (GTP) or ATP from guanosine diphosphate (GDP) or adenosine diphosphate (ADP), respectively [49]. The electrons conserved in NADH and FADH_2_ are then used to reduce oxygen in the electron transport chain of oxidative phosphorylation. (**D**) Oxidative phosphorylation. Occurs across the mitochondrial membrane. Subunit complexes (I–IV) and two electron transfer molecules, ubiquinone (Q) and cytochrome C (C) form the electron transport chain along the inner mitochondrial membrane. Electrons, generated form the reduction of proton donors (NADH and FADH_2_), are transported along the chain to oxygen (O_2_) where they produce water (H_2_O). During this process, protons (H^+^) are transported into the intermembrane space, creating an electrochemical transmembrane proton gradient [50]. ATP synthase (V) then transports these protons back to the mitochondrial matrix and utilises the proton-motive force to synthesise up to 34 molecules of ATP from ADP [50]. Figures created with BioRender.com (accessed on 25 November 2022) and largely adapted from figures created by Amaral [49] and Visconti [42].

Glycolysis is the process of glucose being converted into pyruvate (Figure 3A). During this process energy is released in the form of ATP and nicotinamide adenine dinucleotide (NADH) at a rate of 2 ATP molecules per glucose molecule [40]. Glycolysis is less efficient than oxidative phosphorylation in terms of number of ATP molecules generated, however it produces ATP within the subcellular compartment where it is most needed to fuel motility. It is important to note that sperm can metabolize other substrates, besides glucose, through the glycolytic pathway to sustain motility. Fructose, which is abundant in seminal plasma, can be phosphorylated by hexokinase, resulting in products (either fructose-6-phosphate or fructose-1-phosphate) which can be utilised during glycolysis [41]. Additionally, sorbitol, a constituent of seminal plasma and uterine fluid, can be converted into fructose by the enzyme sorbitol dehydrogenase [41]. Each step of the glycolytic pathway within spermatozoa is catalysed by sperm-specific isoenzymes (Figure 3A) [42]. Experiments have been undertaken using mouse knockout models, where some of these enzymes (including Phosphoglycerate Kinase, Glyceraldehyde 3-phosphate dehydrogenase, Enolase, and lactate dehydrogenase) were deleted via gene targeting, and all treated individuals presented with either male infertility or subfertility, as well as sperm with motility defects and reduced ATP levels [43,44,45,46]. Furthermore, incubation of spermatozoa with glycolysis inhibitors has been shown to be detrimental to sperm motility [47,48]. As such, energy production via glycolysis is evidently required for mammalian sperm function.

Pyruvate, the end product of the glycolytic pathway, is subject to two possible outcomes dependent on cellular oxygen availability. Under anaerobic conditions, pyruvate is converted into lactate to produces 2 NAD^+^ molecules, which can be reused in the glycolytic pathway and sustain additional ATP production [35]. Alternatively, under aerobic conditions, pyruvate is imported into mitochondria by specific transporters [51] and oxidised into acetyl coenzyme A (acetyl CoA) which enters the Krebs cycle by the pyruvate dehydrogenase complex [52,53]. The Krebs cycle, also called the citric acid cycle or tricarboxylic acid cycle, occurs in the mitochondrial matrix (Figure 3B) catalysed by sperm-specific isoenzymes, reduces substrates used to fuel the electron transport chain in oxidative phosphorylation including NADH, flavin adenine dinucleotide (FADH_2_) and guanosine triphosphate (GTP) or ATP (Figure 3C). Acetyl CoA used in the Krebs cycle can also be generated by other sources, such as fatty acid beta-oxidation and ketone body catabolism [49,54,55,56], however these mechanisms are not discussed in detail in this review.

Oxidative phosphorylation, the most efficient ATP generating pathway, is an electron transfer chain driven by substrate oxidation in the mitochondria (Figure 3B), which synthesises ATP through an electrochemical transmembrane gradient (Figure 3D) [57]. The reducing equivalents (NADH and FADH_2_) required for oxidative phosphorylation are provided by a combination of metabolic pathways (primarily the Krebs Cycle as well as, fatty acid beta-oxidation and ketone body catabolism) and are introduced into the respiratory chain via three mitochondrial shuttles (lactate/pyruvate, malate/aspartate and the glycerol-3P shuttle) [35,58]. Embedded in the inner mitochondrial membrane there are four multi-subunit complexes (I–IV) and two electron-transfer molecules (ubiquinone and cytochrome C), which transport electrons donated from substrates formed by other metabolic pathways in the form of NADH and FADH_2_ to create an electrochemical gradient of protons across the inner mitochondrial membrane [50]. ATP synthase (complex V) then transports protons back to the mitochondrial matrix and uses the proton-motive force to synthesise ATP from adenosine diphosphate (ADP) [50]. A mature mammalian spermatozoon contains approximately 72–80 mitochondria which are exclusively located in the midpiece of the sperm flagellum [59]. Theoretically, these mitochondria have the capacity to produce more than 30 ATP molecules per glucose molecule [60]. However, it should be noted that this metabolic pathway is dependent on oxygen availability and the ATP produced in the midpiece needs to be exported to the whole tail where they can be used to fuel sperm motility. Similarly to glycolysis, experimental evidence has demonstrated the necessity of oxidative phosphorylation for mammalian sperm motility and function. Incubation of human sperm with electron transport chain inhibitors results in reduced ATP levels and decreased motility [61,62].

It is thought that mammalian sperm cells switch between these metabolic pathways for ATP production depending on oxygen availability and the concentration of substrates (glucose, pyruvate, lactate, sorbitol, glycerol, and fructose) in its surrounding environment [41,48,60,63,64,65]. This allows spermatozoa to adapt their bioenergetic metabolism based on the metabolites available in its environment to sustain motility throughout its journey from the epididymis through the female reproductive tract to the oocyte.

The dominant metabolic pathway for spermatozoon energy generation in vivo appears to be regulated by the conditions in the oviduct of the conspecific female. Thus, energy production varies between species. Human, mouse and boar spermatozoa are predominantly dependant on glycolysis for the production of ATP [45,48,66], while bull, stallion and ram spermatozoa depend primarily on oxidative phosphorylation for ATP generation [67,68]. Further, spermatozoon energy production which is predominantly dependent on oxidative phosphorylation, like that of the ram, appears to be correlated to more rapid sperm motility. For example, stallion spermatozoa which depend on oxidative phosphorylation for normal motility, have almost double the velocity when compared with human spermatozoa which are highly dependent on the glycolytic pathway for ATP generation [68].

### 3.2. Motility Signalling Pathways

During their journey from the epididymis to the oocyte, spermatozoa undergo changes in motility triggered by changes in the extracellular ionic and biochemical environment [29]. Activation of sperm flagellar motility involves activation of both energy metabolism and the axoneme. Calcium pathways and protein kinase A (PKA) pathways are both important metabolic pathways involved in this process of sperm motility regulation [69,70]. Several calcium-permeable channels have been identified in mammalian sperm including cation channels of sperm (CatSper), voltage-gated calcium channels, cyclic nucleotide-gated channels, and transient receptor potential channels [36]. Calcium is a fundamental regulatory factor for sperm capacitation, hyperactivation, and acrosome reaction [36]. Suarez, et al. [71] demonstrated that when intracellular Ca^2+^ concentrations are low sperm exhibit symmetrical flagellar beating, whereas when Ca^2+^ concentrations rise, the flagellar waveform becomes more asymmetric, and sperm become hyperactivated. Furthermore, calcium is involved in regulation of dynein driven microtubule movement through interactions with protein kinases and calmodulin (calcium sensor localised in the sperm axoneme) [72].

Adenylyl cyclases, which can be classified as either soluble or transmembrane enzymes, catalyze the intramolecular cyclisation of ATP to cAMP through the release of pyrophosphate to control intracellular cAMP concentrations [73]. Soluble adenylyl cyclases are directly activated by bicarbonate and calcium and play a critical role in cAMP signaling by acting as a sensor for ATP, Ca^2+^, and HCO_3_^−^ at various intracellular locations [73,74], whereas transmembrane adenylyl cyclases are mainly regulated by heterotrimeric G-proteins and are involved in motility activation through cAMP-dependent protein phosphorylation [75].

Regulation of sperm motility begins with activation of sodium bicarbonate cotransporters (Na^+^/HCO_3_^−^) which increase intracellular HCO_3_^−^ levels [36]. Concurrently, the activation of sodium-hydrogen exchangers (Na^+^/H^+^) increase the intracellular pH and, in combination with extracellular progesterone, activate CatSper channels which subsequently increase the internal Ca^2+^ concentration [76,77]. Increased levels of intracellular bicarbonate and calcium activate soluble adenylyl cyclases, inducing the conversion of ATP into cAMP which in turn promotes capacitation and the acrosome reaction and, activates serine/threonine PKA pathways [78,79]. Activation of serine/threonine PKA induces a cascade of protein phosphorylation events involved in sperm motility, including phosphorylation of axonemal dynein arms and tyrosine phosphorylation to prepare the capacitated sperm for fertilization [79]. These phosphorylation events are induced as a result of activated PKA regulatory proteins binding to A-kinase anchoring proteins (dominantly, AKAP3 and AKAP4 structural proteins in the fibrous sheath), which promotes tyrosine phosphorylation and indirectly activates Tyrosine kinase [37,80,81,82].

Potassium channels also play an important role in regulating sperm motility by controlling cell membrane potential which influences the activity of voltage-sensitive ion channels [83]. Further, potassium channel activity appears to regulate sperm function indirectly through controlling voltage-gated calcium channels, increasing of calcium concentration in non-capacitated and capacitated cells [84,85]. Further information on the specific signalling pathways and membrane channels involved in each of these processes is beyond the scope of the current review, but can be found in excellent reviews by Inaba [29], Pereira, et al. [36] and, Vyklicka and Lishko [86].

### 3.3. Mechanics of Movement

The axonemal dynein arms have a role in regulating motility by promoting sliding of a microtubule doublet in relation to adjacent flagellar filaments. The flagellar beating pattern begins with ATP being utilised in the midpiece. Na^+^/K^+^ ion pumps in the plasma membrane exchanges three cytoplasmic Na^+^ for two extracellular K^+^ using energy from the hydrolysis of ATP [87]. This creates an electrochemical gradient which drives transport of proteins which induce movement, across the plasma membrane [87]. Subsequent protein phosphorylation, regulated by the radial spokes and central pair, induce the axonemal dynein to move towards the base of the flagellum in an ATP-dependent manner, forcing the attached microtubule doublet to slide down [88]. As they are connected through the nexin links, the dynein from one doublet interacts with the following doublet, and so on. The attached microtubules encounter resistance which converts the interdoublet sliding into axonemal bending [89,90]. Sale and Satir [89] demonstrated that the direction of this sliding process is uniform around the axoneme. Dynein activity switches regularly in such a way that the dyneins on one side of the axoneme are activated, bending the flagellum in one direction, while the dyneins on the opposite side are inactive, generating the characteristic flagellum waveform movement [89,91]. The modulation of this flagellar bend is not yet fully understood, however dynein arm activity, and consequently sperm motility, appears to be modulated by a number of factors, including alterations in pH, ATP availability, calcium concentration, and phosphorylation of key proteins as described previously [40].

## 4. Factors Affecting Sperm Motion Characteristics

### 4.1. Morphology and Species Variation

Although mammalian spermatozoa are consistent in their general structure, a great deal of morphological variation exists between species, specifically in the shape of the head, the length of flagellar segments and, the size of accessory structures including axonemes, outer dense fibres, mitochondrial sheath and fibrous sheath [10]. Further, these variations in sperm micro- and macro- morphology appear to influence differences in sperm metabolism and velocity between species [9,10,24]. Dresdner and Katz [9], demonstrated a linear relationship between the swimming speed of a spermatozoon in a viscous environment, and its flagellar length. Further, the rate at which hydrodynamic energy is expended by the cell, increases with the square of the beat frequency and the cube of sperm length [9]. Consequently, larger mammalian sperm, such as those of the hamster, swim faster and expend significantly more hydrodynamic energy in comparison to relatively smaller spermatozoa, such as those of the ram and human [9].

The lengths of flagellar segments, in particular the midpiece and principal piece, appear to be critical in influencing sperm motility and fertilisation capacity [9,10,92,93,94,95]. In mammalian species, the length of the connecting piece and end piece are typically short, whereas, notable variation in flagellar length between species can be attributed to the length of the midpiece and principal piece [96]. Additionally, sperm movement is driven by a combination of ATP production by the mitochondria (contained in the mitochondrial sheath found in the midpiece) and glycolysis (which occurs in the fibrous sheath found in the principal piece) and, subsequent movement of the axonemal dynein in the midpiece and principal piece [58]. Mitochondrial volume has been associated with sperm length and flagellar beat frequency in 10 mammalian species. Gu, et al. [10] demonstrated that spermatozoa with larger midpieces, and predictably greater mitochondrial volume and function, swim faster (compared using CASA measurements of straight line velocity) than spermatozoa with shorter midpieces. However, in this study, although ATP content is slightly positively correlated with swimming velocity, the correlation coefficient between mitochondrial volume and swimming velocity was significantly smaller than the correlation coefficient between mitochondrial volume and ATP content, suggesting that other factors also impact swimming velocity [10].

### 4.2. Cell Damage

As previously mentioned, the motility of a spermatozoon is dependent on the amount of energy generated by a combination of metabolic pathways, the Krebs cycle and oxidative phosphorylation in mitochondria and glycolysis which occurs in the fibrous sheath [40,49]. As such, sperm cell damage which affects the mitochondria, cell membrane and/or fibrous sheath in the principal piece of the flagellum, result in altered sperm motility by reducing the cell’s capacity for ATP production.

Oxidative stress is a condition experienced by spermatozoa that occurs when the production of Reactive Oxygen Species (ROS) overwhelms the cells antioxidant defenses [97]. Low and controlled concentrations of ROS, produced primarily as a by-product of cell metabolism, are necessary for normal sperm physiological processes such as capacitation, hyperactivation, acrosome reactions and signalling pathways [98]. ROS contain at least one unpaired electron, making them very reactive species, which bind with sperm biomolecules and damage the cell in the form of peroxidative damage to the sperm plasma membrane, DNA damage and triggering of cell apoptosis [99]. Lipid peroxidation in particular, results in impaired motility by interfering with the cells motility signalling and metabolism pathways and decreasing axonemal protein phosphorylation, resulting in sperm immobilization [98]. In comparison to other species, ram spermatozoa are more sensitive to oxidative stress as they contain higher amounts of polyunsaturated fatty acids susceptible to ROS insult [100,101].

Cryopreservation is a process of long-term cell preservation which includes freezing and storage at cryogenic temperatures (−196 °C) and subsequent thawing of cells, with the aim of maintaining cell viability. Cryopreservation generates major advantages for the ovine artificial reproduction industry including the facilitation of international semen transport, prolonged storage of desirable genetic material and increased control over the timing of insemination and thus, birth of the offspring. However, despite advancements in the cryopreservation process, including improved cryoprotective agents and optimisation of the rates and temperature at which freezing and thawing occurs, reduced motility is characteristic of ram spermatozoa following cryopreservation [102,103,104,105]. During cryopreservation, cells are exposed to serve osmotic stress, extreme temperature changes and intracellular ice crystallisation, which induces cellular damage in spermatozoa including changes in cell plasma membranes, nucleus, mitochondria, acrosomes and axonemes [103,104,106,107,108]. Consequently cryodamage alters the cell membrane structure as well as the cell’s metabolism and mitochondrial bioenergetic processes [107,109,110].

Using human sperm, O’Connell, et al. [107] assessed mitochondrial function and sperm motility before and after cryopreservation using Rhodamine 123 (R123) uptake and intensity and CASA motility assessment, respectively. It was found that all CASA motility parameters (except amplitude of lateral head displacement) discussed later in this review, were similarly reduced and that the reduction in the number of viable spermatozoa (viability: 31%) was nearly identical to the reduction in the number of spermatozoa with functional mitochondria (R123 uptake: 36%) [107]. These results suggest that the reduction in mammalian sperm motility caused by cryodamage can be largely attributed to impaired mitochondrial function.

### 4.3. Maturation Stage

As spermatozoa move from the epididymis towards the oocyte, they undergo maturation processes which induce changes in motility. These changes improve the effectiveness of their migration through the female reproductive tract and assist in achieving successful fertilisation.

Spermatozoa acquire activated motility in the male reproductive tract during transit through the epididymal duct. This initial motility development involves the spermatozoon acquiring the potential for flagellation followed by the coordination and modulation of the flagellar waveform, enabling the characteristic swimming pattern [111]. Activated motility is characterised by low amplitude symmetrical tail movements which allow spermatozoa to be progressively motile in low viscosity media, like seminal plasma [112].

In the female reproductive tract, spermatozoa undergo capacitation and hyperactivation in preparation for fertilization. Capacitation involves biochemical changes in the plasma membrane of the sperm head, that reduces binding affinity for the oviduct epithelium and prepare sperm to undergo the acrosome reaction and fertilize oocytes [113,114]. Hyperactivation is a part of the capacitation process that occurs in the oviduct which alters flagellar motion by increasing flagellar bend amplitude and beat asymmetry [115,116]. Thus, hyperactivated motility is characterised by high amplitude and asymmetric flagellar bends which induce an irregular or highly curved trajectory in low viscosity media [13]. However, in viscous media, similar to the oviduct environment, hyperactivated spermatozoa have a relatively straight trajectory [28].

Although the exact physiological basis for it is still unknown, hyperactivation appears to enhance the ability of spermatozoa to migrate through viscoelastic substances and enables penetration of the extracellular matrix of the cumulus oophorus and zona pellucida of the oocyte, to allow the genetic information contained in the sperm head to fuse with the oocyte plasma membrane [1]. Hyperactivated sperm have an improved ability to move through artificial mucus such as viscoelastic solutions of long-chain polyacrylamide or methylcellulose in comparison to non-hyperactivated sperm [117,118]. Furthermore, when hyperactivation was interrupted in capacitated, acrosome-reacted hamster sperm bound to the zona, they were unable to penetrate it, suggesting that hyperactivation facilitates penetration of the mammalian zona pellucida [119].

### 4.4. Environment

Throughout their journey from the epididymis to the oocyte in the female reproductive tract, mammalian spermatozoa encounter different physiochemical environments that influence their motility. Ram spermatozoa must navigate through the complex fluidic and biochemical environment of the ewe reproductive tract including the: vagina, cervix (unless bypassed by artificial means), uterus, uterotubal junction and fallopian tubes (oviduct) [1]. Similarly, the environment sperm are exposed to in vitro during semen assessment or processing has an effect on motility.

#### 4.4.1. Temperature

In nature mammalian spermatozoa spend their entire life span within the male and female reproductive tract and are therefore constantly maintained at body temperature (37 °C). Makler, et al. [12], demonstrated that human sperm velocity increased steadily from zero to 50.4 nm/sec between freezing point (0 °C) and body temperature (37 °C), with activity dropping dramatically thereafter and total immobilization of cells occurring at 45 °C. Based off these findings, it is suggested that high temperatures increase activity and consequently shorten survival time by exhausting resources available for cell metabolism [12]. Furthermore, temperature-related phase transitions of membrane lipids (from the gel to the liquid-crystalline state) that occur at lower temperatures, reduce cell motility by slowing the activity of Na^+^/K^+^ ion exchange pumps resulting in accumulation of sodium, and potassium deficiencies within the cell which interfere with normal cell metabolism and function [12]. Ram sperm were found to experience interruptions in calcium transport resulting in reduced ATPase activity, at temperatures of 23–26 °C and, intramembrane lipid scattering resulting in membrane particle redistribution/aggregation at 17 °C [11].

#### 4.4.2. pH and Osmolarity

In nature, ram spermatozoa in combination with seminal fluid are deposited into either the vagina, cervix or uterus, where it comes into contact with cervical mucus and subsequently progresses into the uterine cavity and fallopian tubes [120]. Within these areas, spermatozoa encounter changes in pH and osmolality which alter their motility and viability. Makler, et al. [14] found the pH of fresh human ejaculates to range from 7.2 to 8.2 and the osmolarity to range between 300 to 380 mOsm/kg (milliosmoles per kilogram). Further, adjusting the pH and osmolarity of the media to either side of these values resulted in progressive loss of sperm motility, although sperm velocity was slightly increased by mild alkalinization and hyperosmolarity [14]. The optimum levels of pH and osmolarity for sperm velocity and viability found by Makler, et al. [14], coincided with the respective pH and osmolarity of environments in the female reproductive tract, including the cervical mucus, uterine cavity, and fallopian tubes [121,122,123].

Zhou, et al. [124] similarly found that a pH of between 7.2 and 8.2 appeared to be the optimum range for total motility and progressive motility of human sperm. In addition, Zhou, et al. [124] found that the activity of Na^+^/K^+^ ion exchange pumps significantly decreased in pH 5.2 and 6.2 media, in comparison to the optimum pH 7.2 and 8.2, suggesting that an acidic medium reduces mammalian sperm motility via downregulation of Na^+^/K^+^-ATPase activity. The authors further suggested that in addition to Na^+^/K^+^-ATPase downregulation, acidic environments effects sperm motility via direct damage to sperm cell membranes and an increase in active oxygen content, however these relationships are not yet fully understood and further exploration is warranted [124].

#### 4.4.3. Viscosity

A ram spermatozoon encounters environments of varying viscosities through its journey from the epididymis, through the ewe reproductive tract to the site of fertilisation including, seminal plasma, cervical mucus and oviductal isthmus medium. It is widely accepted that medium viscosity alters aspects of sperm kinematics including flagellar beat frequency, waveform, and trajectory [13,15,25]. Analysis of marine invertebrate spermatozoa revealed that as viscosity increases, propagation velocity, wave amplitude and energy expenditure of the sperm flagellar decreased [125].

Hyun, et al. [15] demonstrated that as fluid viscosity increases, curvilinear velocity of human sperm decreases. The result is expected considering that as mammalian spermatozoa propel through fluidic environments, it is constrained by frictional forces created by the viscosity of the media which must be overcome to facilitate movement (i.e., a high viscosity media proposes a greater fictional force). However, what is interesting is the relationship between fluid viscosity and sperm swimming force, measured using the minimum laser power required to hold an individual human sperm cell in an optical trap [15]. As viscosity increases, the swimming force of sperm increases, however, aerobic energetics remains relatively constant across treatments [15]. It was previously hypothesised that an increase in sperm swimming force could be explained by an increase in sperm energetics, specifically ATP generation, in response to an increase in the viscosity of its environment [15]. Although these results do not indicate a relationship between swimming force and mitochondrial ATP generation, further investigation into this relationship and the role of anaerobic ATP generation (glycolysis) is required to understand the biochemical properties of the flagellum in relation to swimming force and sperm motility.

Furthermore, Smith, et al. [25] assessed mammalian sperm motility in low and high viscosity media and found significant differences between their respective flagellar wave-speed, wavelength and beat frequency, while the mean progressive velocities remained similar across the treatments. A decrease in spermatozoa wave-speed in high viscosity medium (~35%), in comparison to low viscosity media suggests that liquid viscosity constrains the speed of progression of flagellar wave bends, resulting in an increased wavelength [25]. In a high viscosity medium, an increased wavelength appears to increase cell progression per beat and reduce beat frequency, resulting in fewer beats performed per second at an approximately constant speed, and a significant propulsive advantage [25].

## 5. Methods of Motility Assessment

### 5.1. Mass Motility Assessment

Mass motility assessment, or wave motion assessment, assesses the three-dimensional collective wave motion of a group of spermatozoa in an ejaculate. This method of motility assessment is rapid, easy to conduct, inexpensive, and predictive of sperm fertilizing capacity, making it very attractive to ram semen motility assessment in industry [16,126]. This method involves subjectively scoring motility, using a low magnification on a phase contrast microscope, based off the intensity of movement of waves and whirlpools on the edge of a sample of raw semen on a scale from 0 (no motion) to 5 (numerous rapid waves) according to the original method described by Evans and Maxwell [127]. David, et al. [16] found that with an increase in mass sperm motility score of ram ejaculates, lambing rate also increased across all artificial insemination centres studied. These results indicate that mass sperm motility is generally predictive of the fertilisation capacity of individual ram ejaculates [16]. Despite this, the effectiveness of mass motility assessment used for semen quality assurance in industry is limited by its subjectivity [18]. As such, using these methods alone, ejaculate samples can be inappropriately discarded of despite having adequate fertilization capacity, or inversely, retained and utilised in breeding programs despite having poor fertilisation capacity, because of inaccurate scoring. Development of an objective assessment for mass sperm motility would assist semen assessment by minimising variation in scoring as a result of subjectivity, thus reducing inappropriate and inaccurate scoring. Although some attempts have been made to apply mathematical models which assess the hydrodynamic movement of the waves resulting from collective sperm motion to objective mass motility scoring [128,129], little is known about the relationship between these parameters and fertility. Further development of these objective methods and experimental application to determine potential correlations with fertility is necessary to ascertain if this method provides an advantage over traditional subjective mass motility for semen assessment.

### 5.2. Microscopic Motility Estimation

Alternatively, traditional, semen motility assessment involves a technician making a visual estimation of the percentage of individual progressively motile spermatozoa in an ejaculate using a binocular microscope, including phase-contrast and bright-field objectives, an appropriate condenser, and a thermostage allowing examinations at the recommended temperature of 37 °C [23,130]. This method of in vitro motility assessment is labour-intensive and lacks precision, repeatability and accuracy [17]. Variations of between 30–60% have been reported in the motility estimation of the same ejaculates using subjective optical microscopic evaluation [17]. Furthermore, evaluation of sperm motility by eye does not allow precise discrimination of motility differences between samples, rather, only allowing discrete motility scores within increments of 5% [130]. In subjective methods, significant variation arises from differences in skill level, training, and subjective interpretation of individual technicians, making accurate interpretation of results difficult [17].

### 5.3. CASA

Computer-assisted sperm analysis (CASA) technology, first developed in the late 1980s, refers to an automated computational system used for analysis of sperm movement characteristics. CASA is designed to provide accurate and objective information about sperm concentration, viability, dynamics and morphology utilising continuous images of spermatozoa, digital processing and information analysis [131]. Consequently, although many factors (e.g., technician, optics, CASA system, software settings, the number of fields analysed, semen processing, sample concentration, diluent properties and the analysis chamber) can affect motility results, the CASA system has facilitated a more objective assessment of motility parameters. Thus, CASA systems have effectively increased the precision and reliability of sperm motility assessment by providing more information than conventional subjective motility evaluation [19,132]. For this reason, CASA has been increasingly used for assessing sperm quality and function in domesticated animal production laboratories including quality assurance of ram semen marketed for artificial insemination [19,23,116,132,133,134].

Laboratory-based CASA systems common in domesticated animal production laboratories used for semen assessment include: Integrated Visual Optical System (IVOS II) and CEROS II (Hamilton Thorne Research, Beverly, MA, USA), Sperm Class Analyzer (SCA) (Microptic, Barcelona, Spain), AndroVision (Minitube, Tiefenbach, Germany) and Sperm Quality Analyser V (SQA-V) (Medical Electronic Systems, Caesarea, Israel). More recently, however, portable CASA systems have been developed which facilitate on-site semen assessment, allowing for reduced logistical costs and immediate motility analysis. Such technologies include: iSperm (Aidmics Biotechnology Co., Taipei City, Taiwan), Ongo Semen Analyzer (Ongo Vettech, Martonvásár, Hungary), LensHooke X1 PRO CASA System (Bonraybio, Taichung City, Taiwan) and Dynescan (Dyneval, Edinburgh, UK). Although limited information has been published in scientific literature, the Dynescan appears to assess sperm kinematics differently to other CASA systems. Marketing material suggests that Dynescan assesses the amount of light that passes through sample, at what speed and in what pattern it changes, as opposed to other CASA systems which measure motility through the tracking of individual sperm heads. It will be interesting to see how this new method of CASA motility assessment correlates to in-field fertility, in comparison to traditional CASA motility parameters.

Conventional CASA systems measure multiple motion parameters. Using the images obtained from real-time video capture of the ejaculate, the CASA software is able to report total motility (percentage of sperm that exhibit motility of any form) and progressive motility (percentage of sperm that exhibit rapid, linear movement), as well as multiple measures of sperm velocity outlined in Table 1, including: average path velocity (VAP), straight line velocity (VSL), curvilinear velocity (VCL), amplitude of lateral head displacement (ALH) and beat cross frequency (BCR). From these values, additional measurements regarding sperm movement trajectory can be obtained including: linearity (the linearity of actual sperm track, LIN  =  VSL/VCL), wobble (departure of actual sperm track from average path, WOB  =  VAP/VCL), straightness (linearity of the average path, STR  =  VSL/VAP) and mean angular displacement (mean turning angle of the sperm head along the curvilinear trajectory, MAD).

The relationship between sperm motion parameters generated using the CASA system and in vivo fertility has been studied in many species including the ram, as reviewed by Yániz, et al. [132]. The results from these studies are highly variable, ranging from no correlation [135,136] to a significant correlation [5,137] found between sperm motility and field fertility [132]. Interestingly, studies which combined CASA motility parameters as well as other sperm quality traits in multiple regression models usually showed a stronger predictive correlation with fertility in comparison to the use of single traits. This occurrence may be because many of these parameters which impact fertilisation capacity, and as such in vivo fertility, are not accounted for through single CASA motility measurements assessment alone [21,137,138,139,140,141,142].

This correlation between CASA motility analysis and in vivo fertility has been studied relatively less in rams compared with other species but maintains varying results [20,21,22,136,143]. O’ Meara, et al. [136] reported no relationship between CASA analysis of ram semen and in vivo fertility. Contrastingly, Smith, et al. [21] reported a significant correlation of CASA motility parameters (including the percentage of motile spermatozoa, MAD and BCF) as well as changes in these parameters over 6 h of incubation, with field fertility, demonstrating the potential of these parameters as predictors of ram fertility, once site effects have been accounted for. Similarly, Del Olmo, et al. [20] found that several CASA parameters showed a high positive correlation with fertility after 2 h of incubation in freezing extender, including VAP (R^2^ = 0.678), VCL (R^2^ = 0.745) and BCF (R^2^ = 0.852). Vicente-Fiel, et al. [22] also reported that in a group of high fertility rams all CASA motility parameters (except BCF) were found to be significantly higher in comparison to the low fertility group. Furthermore, in vitro investigations have been undertaken to study the relationship between CASA motility parameters and ram sperm migration in ewe cervical mucus [144]. Robayo, et al. [144], found VCL and VAP to be positively correlated with the ability of spermatozoa to migrate through ewe cervical mucus, suggesting that specific motion parameters which facilitate spermatozoa motility through epithelial mucus, may be important determinants for fertilisation and as such, fertility. In summary, although ram field fertility cannot be directly predicted by CASA motility assessment, CASA analysis is able to provide information important for semen quality assessment, and for studying the effect of changes in the microenvironment on specific sperm motion characteristics.

The ability of the CASA to provide accurate motility assessment results is thought to be restricted by biological and technical limitations. The current CASA motility systems use algorithms to obtain spermatozoa trajectory by exclusively examining the pattern of sperm head motion in two dimensions, with some newer systems beginning to check for the presence of a tail to help exclude non-sperm cells from analyses [145]. However, the CASA systems do not yet have the capacity to analyse flagellar beating directly, despite the flagellar being the primary driver of sperm motility [116]. Only the flagellum contains the motile apparatus necessary for inducing and regulating motility of the entire cell, so it follows that analysis of specific flagellar metabolism and motion parameters will provide a more detailed insight into sperm motion and thus, a better assessment of overall motility of the spermatozoon.

### 5.4. Three-Dimensional Sperm Tracing

Although CASA motion analysis which traditionally, exclusively relied on examining the trajectory of the sperm head in two dimensions has been valuable to assess sperm motility, the advantages of three-dimensional sperm motion analysis remain unclear. The understanding of three-dimensional sperm kinematics has been restrained by limitations in lens-based optical microscopes used in most CASA systems, including poor depth resolution and a trade-off between lateral resolution and field of view [146]. As sperm swim in three dimensions through the female reproductive tract to the site of fertilisation, it is thought that visualisation of spermatozoa swimming trajectory in three dimensions may provide a new range of sperm kinematic parameters. It is expected that measurement of these parameters will complement the currently available two-dimensional based assessment methods, to improve the understanding of sperm function, quality, and fertilisation capacity.

Many methods of three-dimensional sperm tracking have been published which predominantly rely on laser technology or holographics to determine a three-dimensional swimming path, that relates closely to traditional two dimensional CASA analysis [147,148,149,150,151]. Su, et al. [149] developed a method for high-throughput three-dimensional sperm tracking on a chip which uses holographic imaging to reconstruct the trajectories of sperm cells with submicron three-dimensional positioning accuracy. This holographic three-dimensional tracking of spermatozoa in large sample volumes could be useful to better understand the biophysics of sperm locomotion [146]. The motility assessment method with significant research potential, developed by Soler, et al. [148] using boar spermatozoa, found some differences in the kinematics when comparing traditional two-dimensional CASA analysis to this new three-dimensional technology. However, the technology required for this method is not commonly available in most semen assessment laboratories and as such, these three-dimensional motility assessment techniques are not currently suitable for commercial semen quality assessment in the sheep industry.

Alternatively, van der Horst and Sanchez [152] developed a simplified model to convert the two-dimensional CASA sperm tracks into three-dimensional swimming trajectories through mathematical determination of the Z-axis. VCL, ALH and BCF were used in this method’s program to calculate the three-dimensional path where VCL = the distance of detailed tracking along each X and Y coordinate, ALH = the width of the helix, BCF = the number of times the track crosses VAP and VCL/(BCF/2ALH) = the distance between peaks of track [152]. However, X and Y coordinates derived from two-dimensional CASA tracking does not take into account the Z-axis of movement of sperm which swim three-dimensionally, and thus the Z-axis is assumed to be harmonic which is often not the case, meaning that although this method is useful for visualisation in a qualitative sense it is not useful for describing quantitative differences in sperm kinematics [152]. Despite the three-dimensional tracks looking superficially quite similar, this technique found that boar spermatozoa with high progressive motility, a low ALH and a high BCF, have a small diameter helix in the resulting three-dimensional visualization [152]. Contrastingly, in Holstein bulls with fast moving sperm, a high ALH and smaller BCF, the resulting three-dimensional track appears more irregular and almost twice the helix diameter [152]. Further experimental investigation into these relationships is necessary to establish which of these patterns relate to high quality sperm and increased fertilization success.

### 5.5. Flagellar Tracing

Computerised analysis of flagellar movement was first applied to semen assessment by Hiramoto and Baba [153], with the development of a software which enabled the semi-automated image capture of sperm flagellar [153,154]. More recently using human spermatozoa, Saggiorato, et al. [155], developed a flagellar analysis approach which involved segmenting out cells and skeletonizing the resulting data to demonstrate that the flagellar waveform is characterized by a fundamental beat frequency and its second harmonic, with the later breaking the symmetry of the waveform to act as a steering mechanism for the sperm cell. Hansen, et al. [156], also developed a software, SpermQ, which quantifies flagellar beat by determining the flagellar trace using time-lapse images acquired by dark field microscopy, and linking beat parameters to the swimming path to describe the location, intensity and local curvature of individual points on the flagellum.

Similarly, flagellar tracing has been applied to ram spermatozoa by Yang, et al. [157]. The approach extracts the shape of the flagellar beat from an image sequence and utilises a tracing algorithm based on a Markov chain Monte Carlo sampling method to obtain flagellar beat patterns [157]. Visual observation confirmed that the sequence generated, aligned with the flagellar in the images, indicating that the proposed method successfully traces the flagellar [157]. Alternatively, novel microfluidic devices have been developed which enable trapping of individual sperm cells and examination of their respective flagellar beat frequency to facilitate analysis of sperm motility in controlled environments [158,159].

All of the aforementioned flagellar analysis approaches require operator intervention for the analysis of each set of imaging data, making these semi-automated approaches only suited to analysis of tens of cells within a reasonable timeframe. Furthermore, the heterogenicity of ram sperm, demands flagellar analysis approaches which can acquire and analyse large quantities of cell data in order to attain the statistical power necessary to draw valid conclusions. As such, although likely useful for research based flagellar analysis, none of these motility assessment techniques are suitable for commercial semen quality assessment.

Gallagher, et al. [160] recently developed software, Flagellar Analysis and Sperm Tracking (FAST), which enables high-throughput analysis of swimming sperm and their associated flagellar beat parameters. FAST’s flagellar waveform tracking and analysis, enables measurement of a new range of flagellar associated motion parameters, outlined in Table 2 [160].

To date, there is currently limited information in literature detailing the relationship between flagellar parameters, such as those FAST generates, and overall cell motility and/or the traditional head motility parameters CASA provides (including VAP, VSL, VCL, and ALH). Flagellar length is the only parameter measured by FAST which has been explored in depth and positively correlated with other motility parameters, including VSL, as discussed earlier in this review [10]. Further, exploration of the significance of the new motility parameters FAST presents and experimental application to domestic species is required before its usefulness in industry can be determined.

### 5.6. In Vivo Motility Assessment

The majority of methods of semen motility assessment used in industry involve the tracking of sperm in artificial environments. However, it is thought that these in vitro measures may not accurately reflect actual sperm transit within the female reproductive tract. In vivo, additional selection pressures, which are not apparent in vitro, alter sperm motion, including three-dimensional movement and sperm guidance mechanisms (namely thermotaxis, chemotaxis, rheotaxis and thigmotaxis) [161]. Despite this, few methods have been developed which have the ability to observe and measure sperm in their natural environment.

Computational measurement utilising Fourier analysis has been applied to the estimation of beat frequency of spermatozoa within the female tract of the neriid fly (*Telostylinus angusticollis*) [162]. This method records high-speed time-lapse videos of in vivo sperm movement and generates a map of beat frequencies by converting the periodic signal of an intensity versus time trace at each pixel to the frequency domain using the Fourier transform [162]. Although further development of this method for semen analysis in humans and domestic species is required, its application may facilitate novel exploration of variation in sperm movement, by allowing measurement of spermatozoa motility in its natural environment [162].

Furthermore, fibered confocal fluorescence microscopy, which provides direct observation of the microscopic structure of living tissue based on Cellvizio technology [163,164] has been applied to facilitate high resolution observation of the in situ motility of spermatozoa in the reproductive tract of the ewe [165]. In this technique, a microscope objective is replaced by a flexible optical fibre miniprobe which is able to obtain images of individual spermatozoa labelled with fluorochromes, R18 and MitoTracker Green FM, while in the female tract [165]. The results from this study, demonstrate the potential of fibered confocal fluorescence microscopy for the study of sperm motility in vivo, within the female reproductive tract, as well as in an artificial three-dimensional environment in vitro. However, development of new analytical methods to determine motility parameters under these new conditions in required before its usefulness in quality assurance can be determined [165].

### 5.7. Molecular Assays for Motility Assessment

As outlined previously, mammalian sperm motility is regulated by a complex assortment of molecular and structural proteins and their associated signalling pathways. Identification and quantification of biological markers involved in motility regulation may serve as an indirect method of assessing sperm motility.

AKAP4 structural protein (an A-kinase anchoring protein which binds to PKA regulatory proteins) is the most abundant constitutive protein in the fibrous sheath of mammalian sperm and have been shown to be a key molecule involved in the regulation of flagellar motion and function [37,166,167,168]. Furthermore, AKAP4 interacts with many protein partners essential in regulation of sperm motility and hyperactivation including kinases, phosphatases, and glycolytic enzymes [80,169,170]. Reduced expression of AKAP4 has been associated with reduced motility and infertility [168,171,172].

As such, proAKAP4, the precursor to AKAP4, has been identified as a promising marker for assessing sperm motility and quality in many mammalian species including the ram [169]. Concentrations of proAKAP4 can be determined using molecular assays like the 4MID Kit (4BioDx, Lille, France). This kit is a quantitative immunoassay based on the sandwich ELISA which includes two monoclonal antibodies specific to the proAKAP4 protein [169]. The effectives of such a molecular approach in assessing motility and predicting long term quality of ram sperm has been reviewed by Carracedo, et al. [169].

## 6. Conclusions

To maximise reproductive success in sheep artificial breeding programs through reliable quality assessment of semen, the motility of ram spermatozoa must be accurately determined. To facilitate this, changes in sperm motility which occur as a result of differences in sperm structure and the surrounding microenvironment must be considered. This review has discussed the specific changes which occur to mammalian sperm motility in response to morphology, cell damage, maturation stage and its surrounding environment. Furthermore, this review has evaluated methods, currently used in or intended for use, in industry for spermatozoa motility assessment. Although mass motility assessment is a technique which is rapid, easy to conduct, inexpensive, and predictive of sperm fertilizing capacity in rams, making it very attractive to ram semen motility assessment in industry, it’s subjectivity risks inappropriate and inaccurate scoring of ejaculates. Development of an objective method of mass motility assessment has the potential to improve the effectiveness and efficiency of motility assessment in artificial breeding centres by maximising the use of semen with a high predicated fertilising capacity and reducing the use of semen with a low predicated fertilising capacity. Microscopic motility estimation based on the percentage of individual progressively motile spermatozoa lacks precision, repeatability, and accuracy. Alternatively, CASA technology, provides an objective assessment of sperm motility. Many of the motility parameters it measures have been linked with high in vivo fertility in rams making it a useful tool for semen quality assessment of semen in industry and for studying the effect of changes in the microenvironment on specific sperm motion characteristics in vitro. However, despite the sperm flagellum being the primary driver of sperm motility in a three-dimensional manner, current CASA systems assess motility exclusively via tracing of sperm head motion in two dimensions. New motility assessment technologies which may complement traditional CASA motility analysis by measuring a new range of kinematic parameters, such as flagellar tracing, three-dimensional tracing systems, in vivo motility analysis, and molecular assay-based motility assessment methods, have been developed in an attempt to improve semen quality assessment. However, experimental application of these programs is necessary to fully understand how the subtle changes in metabolism and movement mechanisms measured, can influence overall sperm motility as it moves through the female reproductive tract, and subsequent fertility. This understanding will allow their potential advantages to semen quality assessment in the ovine industry to be determined.

## Figures and Tables

**Table 1:** Illustration of sperm motility parameters generated by CASA.

CASA Parameter	Illustration	Definition
VCL (μm/s) = curvilinear velocity	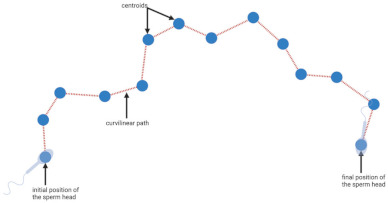	Connecting each centroids * gives the actual trajectory or curvilinear path of the spermatozoon. The time-averaged velocity along this trajectory is termed curvilinear velocity.
VSL (μm/s) = straight line velocity	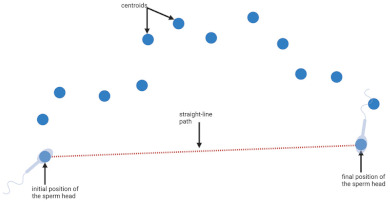	A straight-line path from the first to last position of a sperm head is plotted, and velocity along this trajectory is termed straight line velocity.
VAP (μm/s) = average path velocity	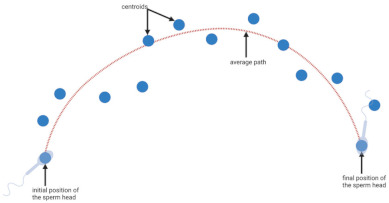	The average path between centroids * is calculated, and time-averaged velocity along this trajectory is termed average path velocity.
ALH (μm) = amplitude of lateral head displacement	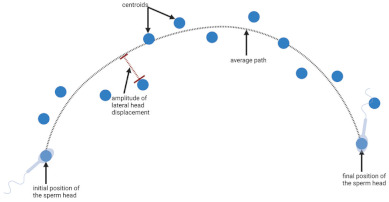	The deviation between each centroid * and the average path, is termed the amplitude of lateral head displacement.
BCF (number per second) = beat cross frequency	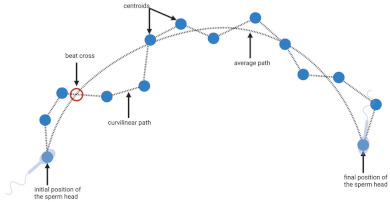	The number of times the curvilinear path intersects the average path is termed beat cross frequency.

* Centroid: generated using image processing based off the position of the head of each spermatozoon in each frame.

**Table 2:** Definition of FAST flagellar parameters and how they are measured.

FAST parameter	Definition
Flagellar beat frequency	Parameters of flagellar waves are determined in terms of their tangent angle; a function of the arclength along the flagellum (measured in micrometers (μm) from the proximal end to the distal) and time. The flagellar beat frequency is calculated by tracking the number of turning points of curvature for a given tangent angle, dividing by the time period and then halving. This is repeated for a number of tangent angles throughout the flagellar to minimize the effects of noise. The median of these values is taken as the flagellar beat frequency of the cell.
Flagellar arc-wave speed	Flagellar arc-wavespeed is measured by calculating the time corresponding to the maximum curvature for each arclength. Iterating forward in arclength, the crest of each wave in time is measured, generating a set of arclength/time pairs for each tracked wave. To fit the wavespeed to these points in a robust manner we formulate a linear mixed effects model (LME) using the generated arclength/time pairs. The output from solving this LME gives a result for arc-wavespeed as well as statistics about the goodness of fit and the random variation within each cells wave speed.
Flagellar arc-wavelength	Flagellar arc-wavelength, measured in micrometers (μm), is calculated by dividing flagellar arc-wavespeed by flagellar beat frequency.
Sperm flagellar length	Flagellar length of a sperm cell is measured form where the connecting piece meets the head to the bottom of the end piece in micrometers (μm).
Flagellar power dissipation (total)	Integrating the hydrodynamic force per unit length exerted on the fluid by the flagellum along its total tracked length, provides a calculation of the energy expenditure (measured in Watts) of the flagellum as it beats. Averaging over the flagellar beat then gives the total flagellar power dissipation of the tracked length of the flagellum.
Flagellar power dissipation (first 30 μm)	Integrating the hydrodynamic force per unit length exerted on the fluid along the first 30 μm of sperm flagellum provides a calculation of the energy expenditure (measured in Watts) of the flagellum as it beats. Averaging over the flagellar beat then gives a comparative power dissipation of each sperm cell, irrespective of tracked flagellar length.
Track centroid speed	Track centroid speed is a measure of the head displacement from the averaged head-track centroid. This is then utilized to differentiate between progressive and non-progressive/immotile cells.

## Data Availability

Not applicable.
